# “Very” Very Late Stent Thrombosis: A Detailed Look at Two Cases

**DOI:** 10.7759/cureus.61914

**Published:** 2024-06-07

**Authors:** Adhvithi Pingili, Shiavax J Rao, Taha Khalid, John Wang, Antony Kaliyadan

**Affiliations:** 1 Internal Medicine, MedStar Union Memorial Hospital, Baltimore, USA; 2 Hospital Medicine, St. Mary's Medical Center, Huntington, USA; 3 Interventional Cardiology, MedStar Union Memorial Hospital, Baltimore, USA

**Keywords:** coronary stent, coronary artery disease (cad), coronary stent thrombosis, st-elevation myocardial infarction (stemi), very late stent thrombosis

## Abstract

Although percutaneous coronary intervention (PCI) has radically transformed the scope of treating coronary artery disease with stenting, stent thrombosis (STh) remains a feared complication. Very late STh, a rare complication after PCI, refers to STh occurring greater than one year after post-stent implantation. An even rarer phenomenon, “very” very late stent thrombosis (VVLST), is described in the literature as STh occurring more than five years post-stent implantation. To our knowledge, there are only 10 case reports and one case series describing VVLST. We discuss two additional complex clinical cases of VVLST presenting as ST-elevation myocardial infarction. We highlight epidemiology, pathophysiology, presentation, diagnostic methods, treatment approach, associated complications, and the need for more extensive future work to minimize the risk of VVLST.

## Introduction

Stent thrombosis (STh) is defined as the angiographic or pathological confirmation of thrombosis within a stent and the presence of one of the three: acute onset of ischemic symptoms at rest, new electrocardiographic changes suggestive of acute ischemia, or typical rise and fall in cardiac biomarkers [1-3]. STh is classified into four types: acute STh (within 24 hours), subacute STh (within one to 30 days), late STh (one to 12 months), and very late STh (VLST) (more than one year) [1,2,4-6]. An even rarer phenomenon, “very” very late STh (VVLST), is described in the literature as STh occurring more than five years after post-stent implantation.

Uncovered struts, stent malapposition or underexpansion, stent edge disease, evagination, neoatherosclerosis, and restenosis are the important pathophysiologic mechanisms of STh [3-6]. Combined analysis of the PESTO (Morphological Parameters Explaining Stent Thrombosis Assessed by Optical Coherence Tomography (OCT)), PRESTIGE (Prevention of Late Stent Thrombosis by an Interdisciplinary Global European Effort), and the Bern registries, which included 401 patients, demonstrated that underexpansion and neoatherosclerosis caused VLST in 23% and 30% of patients, respectively [4-7]. We discuss two intriguing cases of VVLST, which presented as ST-elevation myocardial infarction (STEMI) with angiographic confirmation of STh despite being on antiplatelet therapy.

## Case presentation

Patient 1

**Figure 1:**
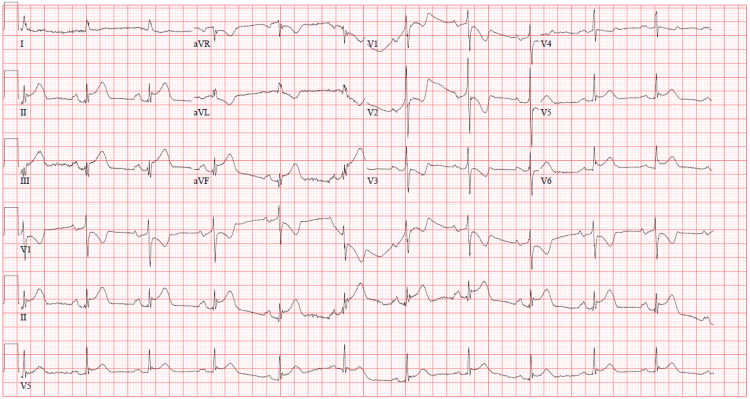
Initial EKG showing acute inferoposterior ST-elevation myocardial infarction.

A 73-year-old man presented to the hospital in the context of chest pain associated with diaphoresis and dyspnea. His past medical history was remarkable for coronary artery disease (CAD) status post coronary bypass grafting (CABG) with a sequential left internal mammary artery sequential graft anastomosed to the left anterior descending (LAD) artery and first diagonal artery eight years prior to presentation. He also underwent subsequent percutaneous coronary intervention (PCI) and stenting of the mid-circumflex artery into the upper first obtuse marginal within a year of bypass grafting. After an initial year on dual-anti-platelet therapy, he then remained on single antiplatelet therapy with aspirin but continued to smoke cigarettes, and he was non-compliant with his lipid-lowering agent. A pharmacologic nuclear stress test performed four months prior to presentation had not shown myocardial ischemia. An initial 12-lead electrocardiogram (EKG) on presentation revealed an acute inferoposterior STEMI (Figure 1).

**Figure 2:**
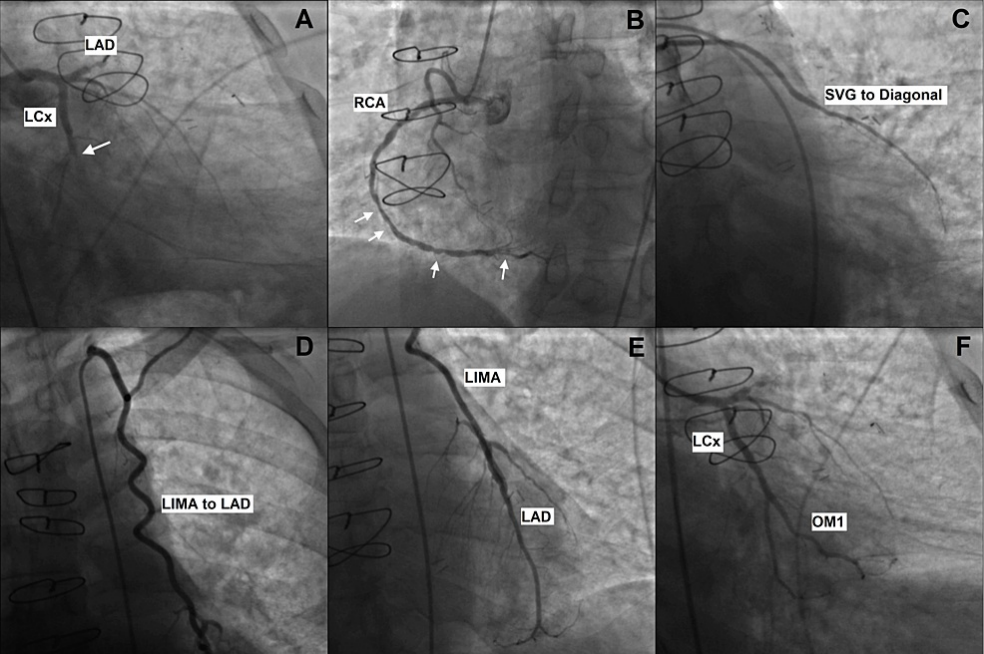
Coronary angiography revealing a right dominant coronary system: (A) LAD with proximal 100% occlusion, large-sized LCx, 100% occlusion of prior mid LCx stent (arrow). (B) RCA with mid and distal 40-50% narrowing, moderate to severe posterior descending artery disease (arrows). (C) Patent SVG to the diagonal artery. (D) Patent LIMA graft to LAD. (E) Widely patent LIMA graft touching down on mid-LAD. (F) Status post-stenting of mid-LCx into OM1 with 0% residual stenosis and TIMI3 flow in the vessel. LAD: left anterior descending artery; LCx: circumflex artery; RCA: right coronary artery; SVG: saphenous vein graft; LIMA: left internal mammary artery; OM1: first obtuse marginal branch; TIMI: Thrombolysis in Myocardial Infarction Score (used to determine the likelihood of ischemic events or mortality in patients with unstable angina or non-ST-segment elevation myocardial infarction)

The patient was loaded with 180 mg of ticagrelor (a direct-acting P2Y12-receptor antagonist), given a heparin bolus, and underwent emergent primary PCI. His coronary artery bypass grafts were patent, but the previously placed stent was found to be jailing both the distal circumflex and the lower branch of the first obtuse marginal, with 100% occlusion (Figure 2). He underwent stenting of the mid-circumflex into the first obtuse marginal, with complete resolution of symptoms. Post PCI troponin-I was 14.2 ng/L (reference range: 0-34 ng/L), and low-density lipoprotein (LDL) level was 220 mg/dL (reference range: 0-99 mg/dL). Transthoracic echocardiography revealed a left ventricular ejection fraction of 60-65%. The patient was discharged on aspirin (indefinitely), clopidogrel (for a minimum of 12 months post-procedure), a beta blocker, and high-intensity statin. He was also counseled on smoking cessation. Dual antiplatelet therapy (DAPT) with clopidogrel and aspirin was continued after consideration of benefits and risks at the one-year follow-up clinic visit.

Patient 2

**Figure 3:**
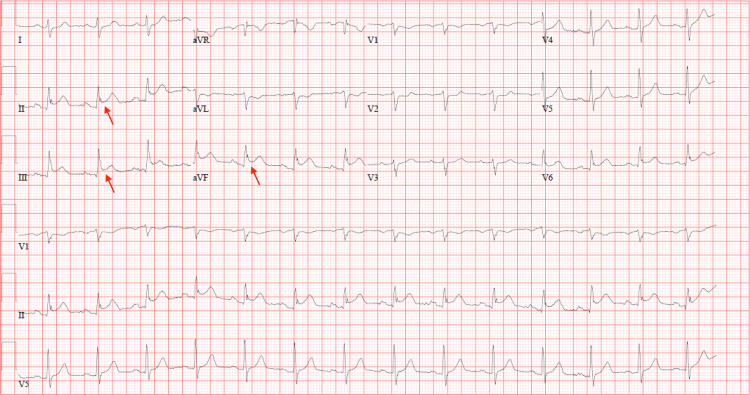
Initial EKG showing acute inferior ST-elevation myocardial infarction with ST-elevations in leads II, III, and avF (red arrows).

A 61-year-old man presented to the hospital in the context of chest pain and dyspnea. His past medical history was significant for CAD status post-PCI, with stenting of the distal circumflex over 10 years prior to presentation. The patient remained on DAPT with clopidogrel and aspirin and lipid-lowering therapy with a high-intensity statin, but he continued to smoke one pack a day. A 12-lead EKG on presentation revealed acute inferior STEMI (Figure 3). He was loaded with ticagrelor, given a heparin bolus, and underwent emergent primary PCI.

**Figure 4:**
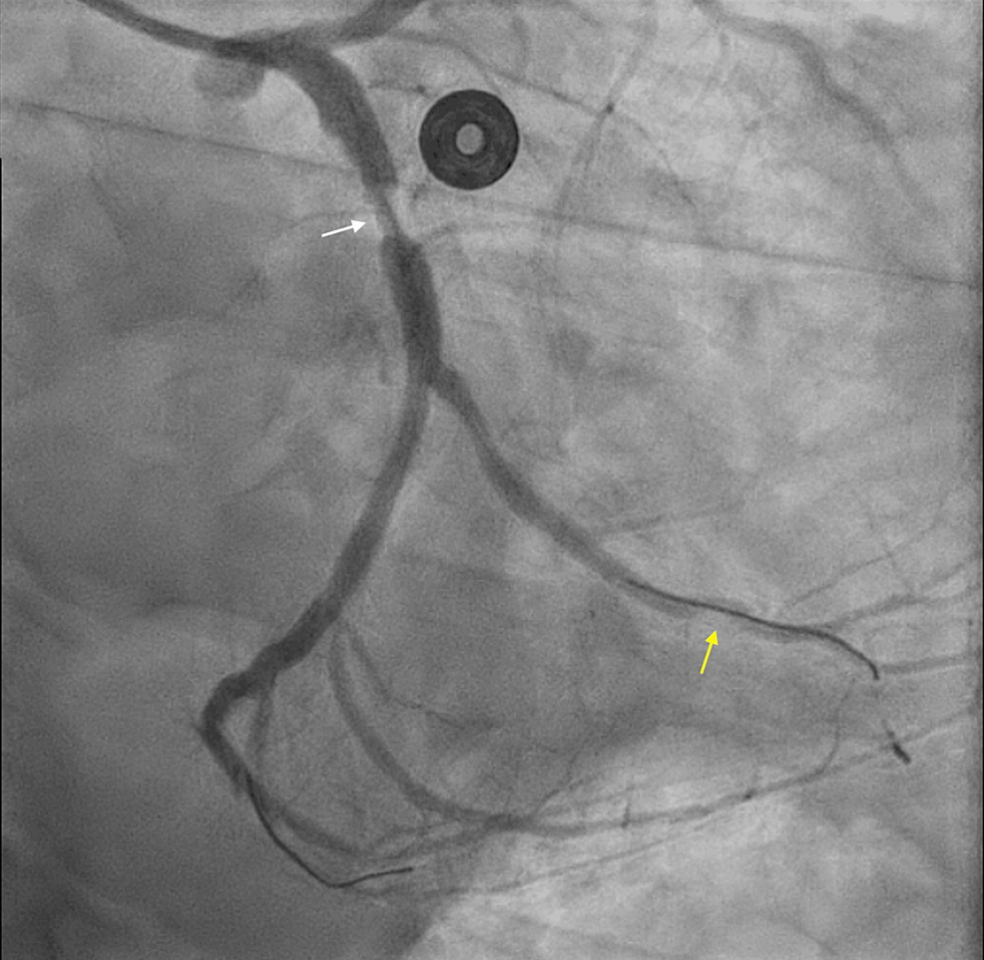
Left heart catheterization revealing 99% occlusion in the previously placed distal circumflex stent (yellow arrow), with 50% stenosis of the mid circumflex and 80% stenosis of the mid LAD (white arrow). LAD: left anterior descending artery

Cardiac catheterization revealed STh with 99% occlusion in the previously placed distal circumflex stent, along with 50% stenosis of the mid circumflex and 80% stenosis of the mid-LAD (Figure 4). He underwent revascularization with a drug-eluting stent (DES) to the distal circumflex and mid circumflex, with complete resolution of symptoms. For the mid-LAD stenosis, staged PCI and stenting were successfully completed. His LDL was 63 mg/dL (reference range: 0-99 mg/dL), and transthoracic echocardiography revealed a left ventricular ejection fraction of 60-65%. The patient was discharged on aspirin (indefinitely), ticagrelor (for at least 12 months post-procedure), a beta blocker, and high-intensity statin. He was extensively counseled on smoking cessation.

## Discussion

The patients in both cases demonstrated ischemic symptoms on presentation, with a 12-lead EKG confirming STEMI. Coronary angiography confirmed thrombus within the prior placed stents for both patients, meeting the diagnostic criteria of STh [1-3], which was ultimately classified as VVLST given the dramatically long intervals between stent placement and thrombosis. Continuation of cigarette smoking, discontinuation of antiplatelet therapy, LDL level greater than 70 mg/dL, diabetes mellitus, and chronic kidney disease (CKD) are important risk factors for STh [3,8,9]. While both patients continued to smoke, patient 1 remained on single antiplatelet therapy with aspirin but was unfortunately non-adherent to atorvastatin, with his LDL level being 220 mg/dL; patient 2 remained on DAPT with clopidogrel and aspirin [10] and was adherent to high-intensity atorvastatin with LDL level being 63 mg/dL. Neither patient had diabetes or CKD. These baseline characteristics in our patients reiterate the importance of risk factor modification in patients to prevent STh.

The mechanism for STh in both patients is thought to be likely neoatherosclerosis, which is characterized by the deposition of lipids and platelet activation [8,11]. Vascular endothelium usually acts as a barrier against the excessive uptake of circulating lipids. However, even moderate levels of LDL cholesterol may lead to the accumulation of lipids in neointima with poor endothelium, resulting in neoatheroscloerosis [8]. In patient 1, the probable triggering factor for neoatherosclerosis was a high LDL level.

Patients with STh have an increased risk of in-hospital mortality, in-hospital major adverse cardiovascular and cerebrovascular events (MACCE), and recurrent STh [3,12]. Intravascular ultrasound (IVUS) and OCT have excellent efficacy in evaluating the mechanisms of STh and, therefore, guiding the treatment [4-7]. Based upon the IVUS or OCT imaging assessment, treatment may involve DES, excimer laser coronary atherectomy, intravascular lithotripsy, plain old balloon angioplasty, or rotational atherectomy, with DES being the most common [4]. Because of the lack of accessibility to IVUS and OCT and time limitations, they were not performed in our patient cases. Nevertheless, given the complications associated with STh, consideration may be given to using IVUS or OCT when managing STh.

Platelet activation is a critical component of the pathophysiology of STh, making DAPT an essential component in preventing STh after PCI [13-15]. In both patients, revascularization was performed using second-generation DES, and they were initially started on DAPT for one year, as well as high-intensity atorvastatin indefinitely. In patient 1, DAPT included clopidogrel and aspirin. However, in patients who are clopidogrel non-responders, ticagrelor or prasugrel should be the drug of choice [16]. The 2021 American College of Cardiology clinical practice guidelines for coronary artery revascularization suggest the continuation of DAPT beyond one year (class 2b recommendation) in patients with minimal risk of bleeding for STh prevention [13]. In addition to reducing the risk of STh, the continuation of DAPT also reduced cardiovascular and cerebrovascular events [15].

## Conclusions

With the advent of second-generation DES, the incidence of STh (particularly VLST and VVLST) has significantly decreased compared to bare metal stents and first-generation DES. However, the risk is still significant enough, given the large number of patients undergoing this procedure. Both cases reinforce the importance of risk factor modification, smoking cessation, continuing high-intensity statin indefinitely, and considering the continuation of DAPT beyond 1 year in patients with a high risk of STh and low risk for bleeding to prevent STh. Furthermore, additional data are needed for the use of IVUS or OCT in STh to better understand the underlying mechanism. This can help guide treatment and improve outcomes in patients with STh.
